# Estimating Volumetric Water Content in Soil for IoUT Contexts by Exploiting RSSI-Based Augmented Sensors via Machine Learning

**DOI:** 10.3390/s23042033

**Published:** 2023-02-10

**Authors:** Matteo Bertocco, Stefano Parrino, Giacomo Peruzzi, Alessandro Pozzebon

**Affiliations:** 1 Department of Information Engineering, University of Padova, 35131 Padova, Italy; 2 Department of Information Engineering and Mathematics, University of Siena, 53100 Siena, Italy

**Keywords:** IoT, IoUT, LoRa, LoRaWAN, virtual sensor, augmented sensor, machine learning, soil moisture sensor, precision agriculture

## Abstract

This paper aims at proposing an augmented sensing method for estimating volumetric water content (VWC) in soil for Internet of Underground Things (IoUT) applications. The system exploits an IoUT sensor node embedding a low-cost, low-precision soil moisture sensor and a long-range wide-area network (LoRaWAN) transceiver sending relative measurements within LoRaWAN packets. The VWC estimation is achieved by means of machine learning (ML) algorithms combining the readings provided by the soil moisture sensor with the received signal strength indicator (RSSI) values measured at the LoRaWAN gateway side during broadcasting. A dataset containing such measurements was especially collected in the laboratory by burying the IoUT sensor node within a plastic case filled with sand, while several VWCs were artificially created by progressively adding water. The adopted ML algorithms are trained and tested using three different techniques for estimating VWC. Firstly, the low-cost, low-precision soil moisture sensor is calibrated by resorting to an ML model exploiting only its raw readings to estimate VWC. Secondly, a virtual VWC sensor is shown, where no real sensor readings are used because only LoRaWAN RSSIs are exploited. Lastly, an augmented VWC sensing method relying on the combination of RSSIs and soil moisture sensor readings is presented. The findings of this paper demonstrate that the augmented sensor outperforms both the virtual sensor and the calibrated real soil moisture sensor. The latter provides a root mean square error (RMSE) of 3.33%, a virtual sensor of 8.67%, and an augmented sensor of 1.84%, which improves down to 1.53% if filtered in post-processing.

## 1. Introduction

The measurement of soil moisture is a key task in a large number of applications in the context of precision agriculture. In fact, autonomous crop watering is included in most precision-agriculture systems, where information concerning soil moisture is one of the main drivers for the activation of irrigation systems. Despite the wide availability of sensors in the market, an accurate measurement or estimation of volumetric water content (VWC) in soil is still a challenging task which requires expensive devices. Indeed, low-cost sensors can easily be found, but they are, in practice, so crude that they are mainly used to distinguish between dry and wet soils. While the latter information may suffice for watering plants in small gardens, it is not sufficiently accurate for precision-agriculture applications, where water usage needs to be optimised to improve overall system efficiency. Indeed, intensive crops may span over thousands of square kilometres, thus requiring pervasive wireless underground sensor networks (WUSN) for remote monitoring [1,2,3,4]. In particular, the interest in such facilities has grown hand-in-hand with the ascent of the Internet of Things (IoT) due to the widespread diffusion of a range of novel communication technologies ensuring long-range coverage, even in underground-to-underground (UG2UG) or underground-to-above-ground (UG2AG) links. As a direct consequence, the concept of the Internet of Underground Things (IoUT) [5,6] has arisen.

The big picture of the IoT depicts low-power wide-area network (LPWAN) technologies as the ones garnering the most attention. Due to their long coverage, up to tens of kilometers, and their limited power consumption, they naturally suit harsh contexts, such as underground. Similarly, their usage is especially fostered by their remarkable robustness, in particular for those operating in sub-GHz bands, (i.e., at frequencies below 1 GHz). Among them, long-range (LoRa) modulation is the one that has undergone the greatest spread. This is due to its exceptional features, the transceivers low cost and high availability on the market naturally eases the deployment of network infrastructures. Thanks to the associated low-power wide-area network (LoRaWAN) MAC-layer protocol, they are capable of handling up to thousands of devices, enabling the set up of pervasive monitoring facilities. In light of this, LoRa-based systems have been extensively adopted in the most diverse application scenarios. Smart City [7] or Smart Industry [8] are examples of the more ordinary ones, but through-metal transmissions for metallic assets monitoring [9], including data transmission from underground for the IoUT [10], represent other, more demanding, cases.

Underground data transmission is especially challenging since the modelling of the quality of the radio channel can be affected by a wide range of different parameters, including the soil composition and soil moisture. In particular, a relation emerged between the level of VWC and radio parameters, such as the received signal strength indicator (RSSI), especially in LoRaWAN UG2AG links [11]. Such relationships are known to allow predictions of the usability of LoRaWAN nodes underground in terms of the maximum nodes burial depth, ensuring reliable communication. More interestingly, they can be inversely exploited, thus gaining other useful information. Indeed, provided that additional data is available (e.g., nodes burial depth, nodes transmitted power output, etc.), radio parameters may be employed to infer the VWC values.

This paper exploits the preliminary results of a previous work [11], which shed light on the correlation between VWC and RSSI in UG2AG LoRaWAN transmissions. In contrast, herein, a machine learning (ML)-based approach is presented. It allows the prediction of VWC values in soil by taking as input both LoRaWAN RSSIs and low-cost, low-precision resistive soil moisture sensor raw readings. Three different techniques are proposed for estimating soil VWC. First, an ML-enabled calibration of the real soil moisture sensor, exploiting its raw readings in order to estimate VWC, is presented. Second, a fully virtual VWC sensor, where no real sensor readings are recorded because only LoRaWAN RSSIs are exploited, is devised. Third, an augmented VWC sensing method, relying on the combination of RSSIs and soil moisture sensor readings, is shown. Although the usage of a coarse sensor may seem unsuitable, it can provide performances comparable to high-accuracy probes whenever it is included within the augmented sensor due to the simultaneous exploitation of RSSIs, yet at a notably much lower cost. While the results presented in this paper are based on a dataset acquired by making use of a single typology of soil (i.e., pure sand), the methodology presented herein can be easily extended to different typologies of soils; in such cases, an initial system parameter tuning phase may be required, entailing the collection of a new dataset related to that specific soil. Despite this initial setup, which is, however, needed in most data-driven approaches, the technique described in this paper can be fully replicated. An alternative way to infer VWC in the case of different soils may be based on the exploitation of the theoretical soil attenuation models, which assumes soil composition as one of the parameters. As a consequence, an accurate soil composition characterisation is needed. Such an operation may be more complex than a simple initial tuning stage, however, and can lead to unreliable results because theoretical models necessarily require the precise soil composition in order to provide consistent outcomes. For this reason, this solution falls outside the scope of this paper. Nonetheless, an overview of the theoretical model is given for the sake of completeness. The main contributions of the paper are the following:

- Proposal of a set of ML-based methodologies for the estimation of VWC exploiting a WUSN enabled by the LoRaWAN protocol;
- Presentation of a threefold sensing technique for VWC, which, respectively, are the calibration of a real cheap soil moisture sensor (that is not suitable for measuring VWC) exploiting its raw readings, the realisation of a fully virtual sensor estimating VWC from LoRaWAN RSSIs, and the implementation of an augmented sensor for VWC, taking as input both the soil moisture sensor raw readings and the LoRaWAN RSSIs;
- Introduction of a filtering process to enhance the performances of the aforementioned sensing techniques.

The rest of the paper is organised as follows: Section 2 discusses the state-of-the-art. In Section 3, the theoretical model regarding LoRaWAN attenuation in soil is presented, while Section 4 is devoted to a description of the experimental setup. Section 5 describes the data-processing techniques, while Section 6 shows and discusses the tests results. Finally, Section 7 highlights the conclusions and presents final remarks.

## 2. Related Work

The LoRa modulation and LoRaWAN protocol are widely adopted amongst a plethora of applications due to their robustness, reliability and wide operating rage. Industrial monitoring and “smart cities” are common application contexts, and harsh environments, such as marine [12] or underground [13,14] situations, are considered in the literature. In particular, the latter is especially critical due to the intrinsic attenuation properties of soil, resulting in hindrance to electromagnetic wave propagation [15,16,17].

It is well-known that propagation in radio communication systems depends on characteristics of the medium. For instance, [18] analyses, by means of simulations, a dipole antenna in the ultra-high frequency (UHF) band (which includes the operating frequencies of LoRa) buried within wet soil. Simulation results showed inverse relationships between VWC, operating frequency and bandwidth, leading to a modification of the antenna radiation diagram making it more directive. Similarly, the dependence of transmission performances within the sub-GHz spectrum (i.e., the one in which LoRa modulation works) on soil characteristics was studied in [19] via computer simulation. Moreover, the dependence on soil moisture of transmission performances for LoRa underground links was investigated by means of simulations in [20], showing that other state-of-the-art competitor cellular technologies (e.g., NB-IoT) may, theoretically, work better than LoRa. Unfortunately, however, the drawback of high running costs occurs whenever pervasive WUSN relying on cellular standards are set up. Apart from simulations, cellular facilities, such as NB-IoT, were tested and compared to LoRa for UG2AG links, highlighting their potential practicability [21]. In addition, the specific effects of soil on LoRa systems have been considered. For example, the correlation between performance drops, inter-nodal distance and soil moisture was analysed in [22]. Moreover, Refs. [23,24,25] put forth solutions to sense VWC by resorting to soil moisture detrimental effects, relying on a LoRa network whose nodes were buried in strategic spots, thus forming a multidimensional arrangement.

Despite the clear influence of soil moisture on radio transmission performances, a surprisingly limited number of investigations have sought to exploit this link to infer VWC values from measurements related to radio parameters. In [26], the estimation of soil moisture, exploiting radio-wave parameters, is proposed and discussed from a theoretical point of view by means of a global sensitivity analysis. However, while this work is targeted on LoRa technology, no field tests were actually performed. An experimental setup based on arrays of LoRa transmitters is presented in [23,24]. Although these studies propose a layout which is comparable with the one presented in this paper, the soil moisture value is only retrieved by exploiting a mathematical model. Conversely, the estimation of soil water content, and, therefore, VWC, can also be performed by resorting to direct or indirect methods. For instance, among the former, ground-penetrating radar (GPR) systems can be retrieved [27,28]; while, among the latter, remote-sensing strategies can be found [29], such as the solution proposed in this paper. Specifically, ref. [27] performed a laboratory study to assess the viability of high-frequency GPR antennas to sense the water content in soils by examining specimens of loamy sand, clay, and silty loam. Such studies have confirmed that the water content in soil is pivotal in altering the soil dielectric permittivity and, thus, soil attenuation properties. Similarly, in [28] a laboratory study on four soils (i.e., sand, sandy loam, loamy sand and clay) was performed to measure water content by means of GPR instruments, leading to comparable results to those of [27]. With regard to indirect methods, Ref. [29] made use of satellite imagery for estimating soil moisture content, showing that only regional-scale estimates can be obtained rather than pervasive ones, indicating that this method is unsuitable for precision agriculture.

ML techniques have been extensively applied to the prediction of soil moisture levels. However, most of the works in the literature exploit different typologies of datasets to that of the approach presented in this paper. A large number of contributions have focused on the exploitation of remotely sensed data; two comprehensive reviews can be found in [30,31]. The estimation of VWC by making use of ML algorithms was also investigated by exploiting datasets acquired by means of satellites [32,33]. However, such methods are very far from the one presented in this paper because satellite data cannot be continuously sampled and customized according to the specific application requirements. Conversely, low-cost wireless sensors can be easily deployed for continuous monitoring wherever required. Moreover, satellite data predictions are obtained at a geographical level only, while sensor networks can be deployed in specific pre-defined spots, hence providing greater accuracy and a more pervasive approach.

The majority of the works in remote sensing address architectures, communication schemes, protocols, or sensing technologies, while a limited number focus on the actual usage of the collected data. In [34], soil moisture measurements were combined with meteorological data to obtain forecasts of future soil moisture values. In [35], the same prediction was achieved by means of meteorological values combined with soil temperature, while a solution focusing on meteorological parameters was also described in [36]. However, all these proposals are aimed at predicting the future values of soil moisture, while the purpose of the method of this paper is the real-time prediction of relatively accurate measurements from low-cost sensors. Moreover, no reported studies propose the usage of radio transmission parameters as additional data for the improvement of ML algorithm performance. In [37], radio echoes generated by means of ultra-wide-band (UWB) GPR are used to infer VWC values by means of neural networks (NN). However, this strategy is far from being low cost and cannot be considered a deploy-and-forget approach. Therefore, to the best of the authors’ knowledge, the approach presented in this work, focusing on a dataset based on radio parameters acquired by means of buried LoRaWAN nodes, and combined with values sampled with low-cost, low-precision, soil moisture sensors, has never been proposed in the literature. Finally, Table 1 lists key findings from related works.

## 3. Theoretical Analysis

**Figure 1:**
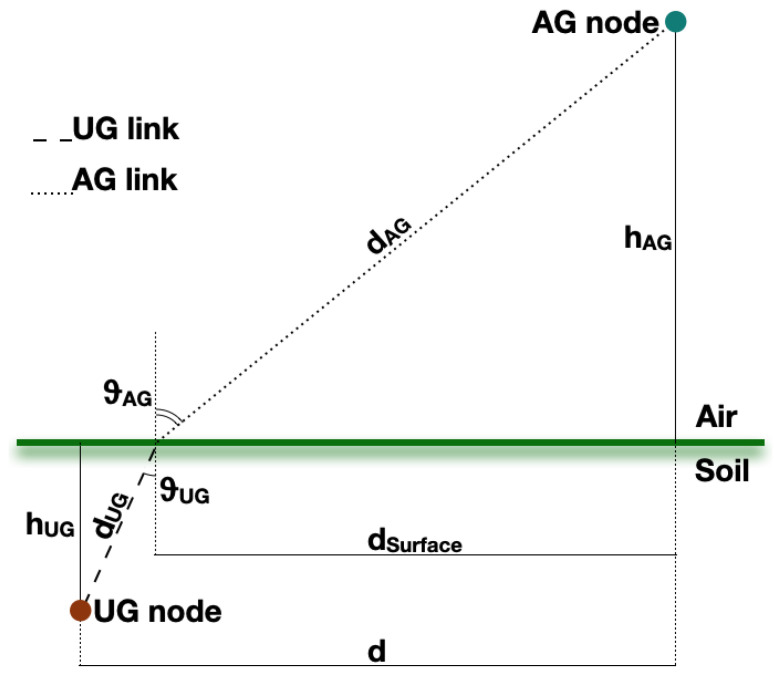
Channel model for UG2AG links.

The concept of path loss is useful to understand the relationships between soil moisture and radio parameters. Relevant literature contributions on underground path loss are found in [38,39,40,41,42,43,44,45,46]. The primary findings of such research are summarised and put into practice herein, bearing in mind Figure 1.

**Figure 2:**
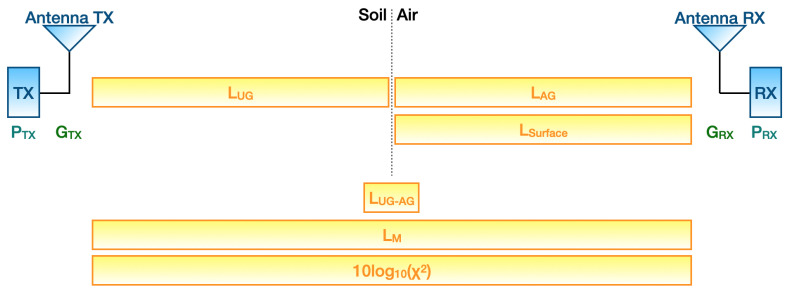
Block diagram of losses and gains occurring during a wireless UG2AG link according to Equation (1).

Path loss can be found from the modified Friis equation:

(1) $$P_{RX}=P_{TX}+G_{TX}+G_{RX}-L_{UG}-L_{UG-AG}-0.5\left(L_{AG}+L_{Surface}\right)-L_{M}-10log_{10}\left(\chi^{2}\right)$$

where $P_{RX}$ is the RSSI, $P_{TX}$ is the transmitted power output; $G_{TX}$ and $G_{RX}$ are, in turn, the gains of the transmitter and receiver antennas; $L_{UG}$ stands for the underground losses; $L_{UG-AG}$ represents refraction losses at the soil-air interface; $L_{AG}$ takes into account aboveground losses; $L_{Surface}$ is the attenuation due to lateral waves; while $L_{M}$ contains miscellaneous losses stemming from sundry sources (e.g., obstacles within the first Fresnel zone, antennas polarization mismatch and so on). Indeed, while the requisite for far-field communication (i.e., implying a distance between the antennas far greater than the carrier wavelength) is mainly satisfied for UG2AG transmissions, antennas having different polarization may be exploited. Moreover, the latter can likely be misaligned since the transmitter antenna is generally housed within an ad hoc box and buried underground. In addition, the burial condition of the transmitter antenna leads to an inability to ensure the hypothesis of unobstructed free space. Finally, $10log_{10}\left(\chi^{2}\right)$ accounts for multi-path fading. Figure 2 shows where each of the components of Equation (1) come into play throughout the wireless UG2AG link.

**Figure 3:**
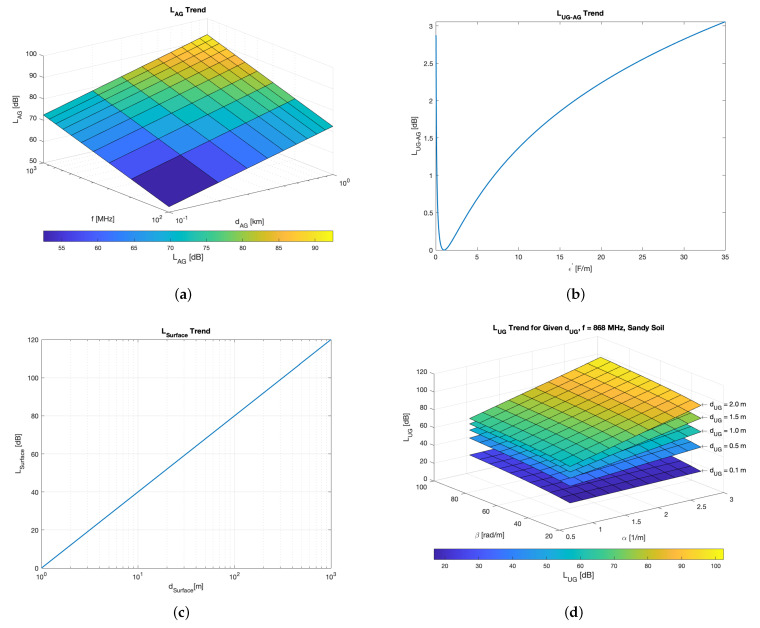
Trend in the losses which are involved in Equation (1): (**a**) $L_{AG}$ (Equation (2)), (**b**) $L_{UG-AG}$ (Equation (3)), (**c**) $L_{Surface}$ (Equation (4)), (**d**) $L_{UG}$ (Equation (5)).

The aboveground losses $L_{AG}$ can be evaluated as free space loss

(2) $$L_{AG}=32.45+20log_{10}\left(d_{AG}\right)+20log_{10}\left(f\right)$$

where $d_{AG}$ is the aboveground link length expressed in km and *f* is the carrier frequency expressed in MHz. This kind of loss strictly depends on the covered distance and the carrier frequency. In particular, a logarithmic dependency arises, as can be seen in Figure 3a. Of course, if, in the application at hand, the hypothesis of free space would not be met, then a proper path-loss model must be considered.

Refraction losses occurring at the soil-air interface are computed according to

(3) $$L_{UG-AG}≃10log_{10}\left[\frac{{\left(\sqrt{\epsilon^{{}^{\prime }}}+1\right)}^{2}}{4\sqrt{\epsilon^{{}^{\prime }}}}\right],$$

while the attenuation due to lateral waves is modelled as

(4) $$L_{Surface}=40log_{10}\left(d_{Surface}\right),$$

where $d_{Surface}$ stands for the length of the soil-air interface. Losses related to the soil-air interface depend on the real part of the effective soil permittivity. As will be explained below, such a quantity strictly depends on the soil composition, the VWC, and the carrier frequency. Its trend is shown in Figure 3b: it has a minimum for $\epsilon^{{}^{\prime }}=1.164$ F/m, and shows logarithmic behaviour for greater values of $\epsilon^{{}^{\prime }}$. On the other hand, the attenuation due to lateral waves, that depends only on the covered distance on the surface in which the two media meet, shows a fully logarithmic trend (see Figure 3c).

$L_{UG}$ may be evaluated by making use of [47,48]:

(5) $$L_{UG}=6.4+20log_{10}\left(d_{UG}\right)+20log_{10}\left(\beta \right)+8.69\alpha d_{UG}$$

where $d_{UG}$ is the underground link length expressed in *m*, while α and β, respectively, are constants for the attenuation and for the phase shifting. The underground losses are highly correlated to the soil composition, owing to the dependence on α and β, and to the carrier frequency and burial depth. In order to provide meaningful insights, Figure 3d shows the $L_{UG}$ trend for f=868 MHz, and for sandy soil. Indeed, the former is the exploited frequency band for the experiments (see Section 4.2), while the latter is the adopted soil for the experiments (see Section 4.1). Figure 3d highlights that, for a given $d_{UG}$, $L_{UG}$ behaves as the superimposition of a logarithmic trend in the function of β and of a linear trend in the function of α. Of course, since a sandy soil and a single carrier frequency are concerned in Figure 3d, variation in α and β can be ascribed to VWC. Indeed, they can be evaluated as

(6) $$\alpha =2\pi f\sqrt{\frac{\mu_{0}\mu_{r}\epsilon_{0}\epsilon^{{}^{\prime }}}{2}\left[\sqrt{1+{\left(\frac{\epsilon^{{}^{\prime \prime }}}{\epsilon^{{}^{\prime }}}\right)}^{2}}-1\right]},$$

(7) $$\beta =2\pi f\sqrt{\frac{\mu_{0}\mu_{r}\epsilon_{0}\epsilon^{{}^{\prime }}}{2}\left[\sqrt{1+{\left(\frac{\epsilon^{{}^{\prime \prime }}}{\epsilon^{{}^{\prime }}}\right)}^{2}}+1\right]}$$

in which *f* is the carrier frequency, $\mu_{r}$ is the relative magnetic permeability of soil, while $\epsilon^{{}^{\prime }}$ and $\epsilon^{{}^{\prime \prime }}$ are, in turn, the real and imaginary parts of the effective soil permittivity. They can be assessed by resorting to two alternative techniques: The mineralogy-based soil dielectric model (MBSDM) [49,50] and the procedures issued by the International Telecommunication Union (ITU) [51].

The MBSDM can be adopted in the range 0.045–26.5GHz and requires the VWC, the carrier frequency and the percentage of clay related to soil composition as inputs. Conversely, the ITU model for path-loss estimation due to soil implies knowledge of the carrier frequency, the temperature, the percentages of either sand and clay, the specific gravity (SG) (i.e., the mass density of the soil sample over the amount of water within it), the VWC and the bulk density (BD) of the soil samples.

Regardless of the model employed in the evaluation of the real and imaginary parts of the effective soil permittivity, in both cases, VWC is one of the parameters to be evaluated. This suggests the reversibility of this relationship to derive VWC from radio transmission parameters, in particular by resorting to channel losses or received power.

## 4. Materials and Methods

In order to gather the dataset, an experimental setup was devised to collect the values from the low-cost moisture sensor, as well as the RSSIs related to the transmitted LoRaWAN packets enclosing the sensor measurements in their payloads. Tests were accomplished in the laboratory, pursuing the aim of controlling the trials and limiting the number of involved variables (e.g., the ones involved in Equation (1)).

### 4.1. Experimental Setup

The dataset was acquired by making use of a plastic case that was filled with sand, while the sensor node was buried within it, thus becoming an IoUT node. The plastic case was a 55×40×30 cm box, containing 66,000 cm${}^{3}$ of sand. The IoUT sensor node was housed within an IP56 box that was positioned with the antenna in the centre of the plastic case. In doing so, the IoUT sensor node had, respectively, 20 cm and 13 cm of sand along the lateral and vertical directions. The soil was utterly composed of sand having a BD of 1.711 g/cm${}^{3}$ and a standard SG value of 2.66 [52].

### 4.2. Hardware Setup

**Figure 4:**
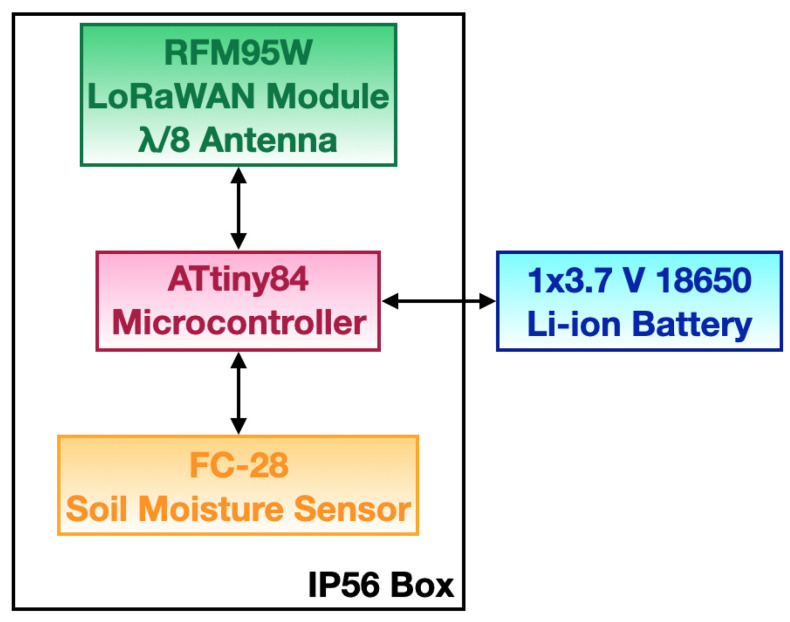
IoUT sensor node block scheme.

The IoUT sensor node (see Figure 4 for its block scheme) was composed of a microcontroller (i.e., Microchip ATtiny84), and a LoRaWAN transceiver (i.e., RFM95 produced by HopeRF) to which a λ/82 dBi-whip antenna was connected. The node was supplied by a 3600 mAh Li-ion battery providing 3.7 V. The electronics, apart from the battery, was housed within the aforementioned IP56 box. The power supply was outside the plastic case and connected to the IoUT sensor node via cable glands passing through the IP56 box. In so doing, the sand moisture setup was not affected during the process of powering up and shutting down the IoUT sensor node. A low-cost (i.e., less than 3 EUR), low-precision analog soil moisture sensor (i.e., FC-28 from Omatom Power) was connected to the IoUT sensor node. It measures soil moisture rather than VWC; accurate VWC sensors are usually very expensive (i.e., up to 100 times more). Such sensors feature a probe with two conductive pads that need to be inserted into the soil. The probe is connected to a board providing an analog output through an embedded amplifier having a range within 0 V and 3.3 V. The output voltage is indirectly proportional to soil moisture, which, in turn, translates into an inverse trend with respect to the soil dielectric constant. When connected to the ATtiny84 microcontroller, which embeds a 10 bit analogue-to-digital converter (ADC), the sensor output voltage is converted into an integer value between 0 and 1023, hereinafter referred to as a “raw reading”, and is exploited by the ML models to infer VWC. However, due to the sensor coarse precision, only two main moisture levels can be clearly identified. In particular, values around 850 correspond to an almost dry to totally dry condition, while values ranging from 300 to 500 correspond to a wet condition (see Section 5 for the detailed analysis). In addition, the microcontroller did not accomplish any additional processing on the raw readings; thus, they were just kept as integer values.

**Figure 5:**
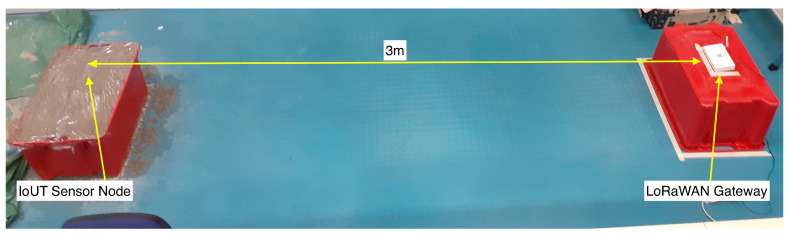
Testing setup: The IoUT sensor node buried in sand within the plastic case and the LoRaWAN gateway.

The firmware running on the microcontroller implemented a Class A LoRaWAN end device, sending packets to a LoRaWAN gateway. The packet payloads contained the raw readings coming from the soil moisture sensor. Such packets were broadcast by exploiting a frequency diversity scheme between 8 different channels belonging to the 863–870 MHz industrial, scientific and medical (ISM) band, and by making use of the minimum spreading factor (SF) provided by the LoRaWAN protocol (i.e., SF = 7) and a bandwidth of 125 kHz. Moreover, the gateway and the buried IoUT sensor node were 3 m apart (see Figure 5) so as to reduce the losses that cannot be ascribed to soil attenuation.

The adopted gateway was an LG308 manufactured by Dragino. Its sensitivity depends on the SF and the exploited bandwidth of the transmitted packets. Therefore, for the aforementioned transmission test parameters, the gateway had a sensitivity of −126 dBm. Moreover, the gateway was provided with the same antenna of the IoUT sensor node (i.e., a λ/82 dBi-whip antenna), and ran a packet forwarder routine. No sooner had it received and demodulated a LoRaWAN packet, than it transmitted it to a remote network server by resorting to the message queuing telemetry transport (MQTT) protocol. The adopted LoRaWAN network infrastructure was directly derived from that described in [53], since the same back-end side was exploited. In particular, the network server consisted of a Node-RED routine that managed all the received packets and stored the related data (i.e., the payloads) and metadata (i.e., RSSIs) within a database.

### 4.3. Methodology

**Figure 6:**
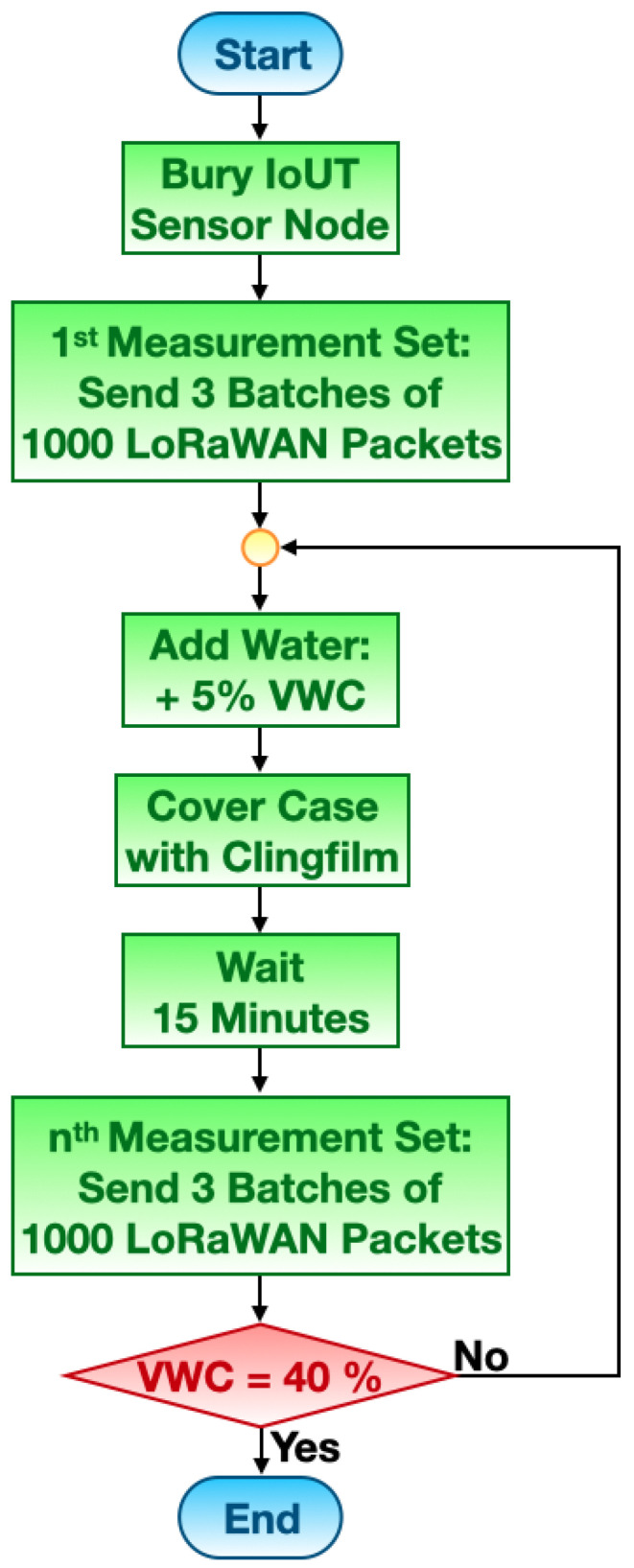
Flowchart of the adopted methodology.

The adopted methodology worked as shown in the flowchart presented in Figure 6, and is described as follows: Each measurement set was composed of three batches, each made of 1000 LoRaWAN packets that were broadcast by the IoUT sensor node, while each of the packets payload contained the soil moisture sensor raw readings. At the receiver side, the gateway made note of RSSIs and of the packet payloads. The first set was characterised by almost dry sand because a minimal amount of water molecules in the sand sample cannot be avoided. Nevertheless, the VWC in this setting can be thought to be equal to 0%. A sandy soil was selected due to its capability of rapidly absorbing water, keeping itself soaked. From the second set on, VWC values were tested with a granularity of 5% by accurately watering the sand. The amount of water to be poured directly stemmed from the definition of VWC. It is specified by the ratio between the volume of water and the unit volume of soil. Due to the dimensions of the plastic case, the sand volume was 66 L. Therefore, augmenting the VWC of 5% directly translated into the addition of 3.3 L of water to the sand. Then, in order to avert water evaporation, the plastic case was covered with clingfilm. No sooner had 15 min passed, then the first batch of transmissions belonging to the measurement set at hand was started. In so doing, the sand had time to uniformly imbibe the water. This procedure was repeated so as to recreate VWC values from 0% to 40%. VWCs above 40% were not tested because the sand had no more capacity to absorb further water. This methodology implies an overall relative uncertainty estimate of the order of ±5% because of the entailed uncertainty related to the measurement of the water and sand volumes.

## 5. Data Processing

### 5.1. Discussion on the Collected Dataset

**Figure 7:**
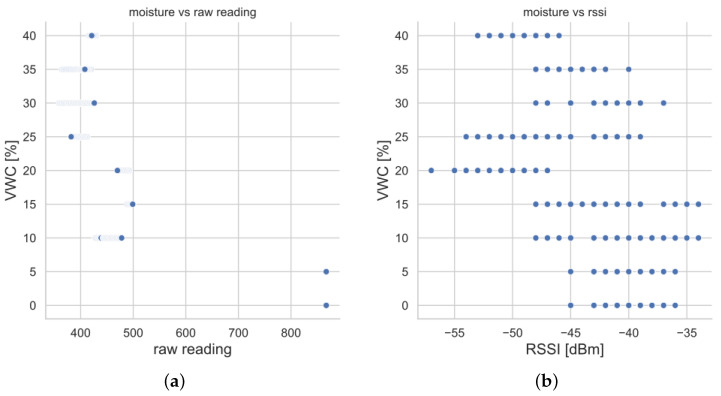
Experimental relationship: (**a**) between collected measured values from the sensor (i.e., “raw reading”) and reference VWCs (i.e., “VWC”), (**b**) between RSSI and reference VWCs (i.e., “VWC”).

The collected dataset during the tests included 27,000 data points. The resulting dataset was exploited to implement several solutions based on ML techniques, aiming at estimating VWC values combining the different typologies of data. First of all, the data was organised in a table, containing the measurements of the low-cost sensor (i.e., raw readings), the RSSI measurements provided by the LoRaWAN gateway, and the reference values for VWC to be exploited as labels. In other words, the dataset labels belong to the set {5i%:i=0,1,⋯,7,8} because they are the reproduced VWCs during the data acquisition phase by watering the sand within the plastic case. Figure 7 shows the experimental relationships between the collected data (i.e., RSSI and raw readings) and the reference VWC labels. Each of the points refer to a LoRaWAN packet; the ones in Figure 7a correlate the labels with the sensor raw readings coded within the packet payloads, while the ones in Figure 7b associate the labels to the RSSIs of the LoRaWAN packets that were measured by the gateway.

The moisture sensor raw readings are very roughly related to reference VWCs due to the coarse precision of the sensor. From Figure 7a, soil moisture can be classified only into a two-valued set: “nearly dry”, where raw readings greater than 800 correspond to VWCs of 0% or 5%; and “wet”, where raw readings less than, or equal to, 500 are paired to VWCs from 10% to 40%. This underlines the fact that the low-cost, low-precision soil moisture sensor only allows discrimination between dry and wet states. Moreover, the “wet” condition can be separated into two further humidity interval sets, but this division is more error prone. Specifically, a “moderately wet” cluster is the one having sensor raw readings ranging from 400 to 500, which are related to VWCs from 10% to 20%; while a “very wet” cluster is composed of points having sensor raw readings less than 400 that are paired to VWCs spanning from 25% to 40%. However, because of the poor quality of the sensor, such clusters overlap, since some points having raw readings in the range 400–430 are associated with high reference VWCs rather than with lower values. On the whole, the measurements provided just by the soil moisture sensor are very rough and do not, in practice, enable achievement of a satisfactory regression curve allowing direct estimation of VWCs starting from such measurements. Conversely, from Figure 7b, it emerges that a clear relationship between RSSI and VWC cannot be found; that is, no bijective functions can be distinguished.

**Figure 8:**
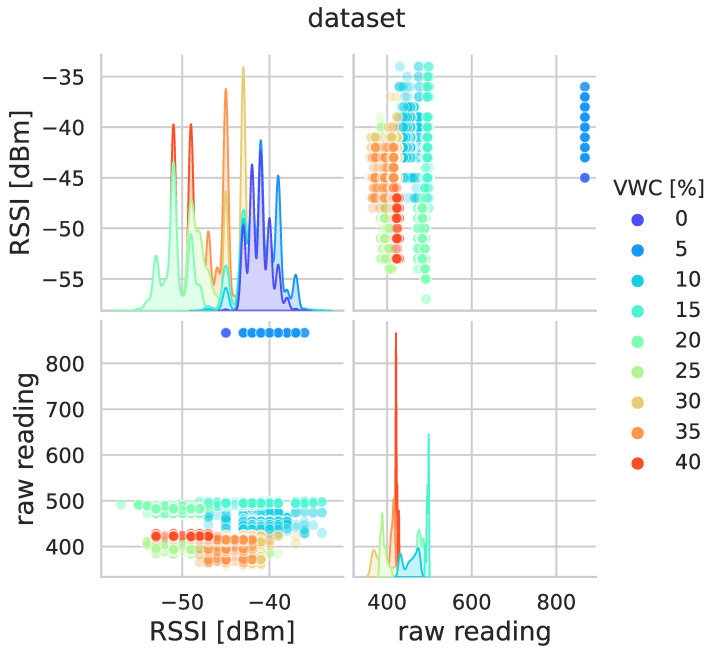
Scatterplots of collected data, showing pair relationships between raw sensor readings and RSSIs. Each pairwise data point is highlighted using a colour code depending on the values of the reference VWC: dark blue represents 0% VWC, up to red representing 40% VWC. Along the diagonal, smoothed histograms using a standard kernel method of the corresponding variable are plotted.

Therefore, simple VWC estimates would be coarse if they only rely on sensor raw readings, or almost unfeasible if they are grounded on the reversibility of the theoretical model taking as input the RSSIs. That is the reason why ML models are exploited. This understanding is also confirmed by Figure 8, which provides scatterplots of the collected data, where the colour codes show that, looking at the various variables in pairs, aggregation of data points arises depending on VWC. This suggests that the mere use of just one variable cannot provide fine VWC estimates, while combination of all the variables may give a more complicated nonlinear relationship with VWC that can be implicitly modelled by ML methods.

### 5.2. Machine Learning Models

Different ML-based regressors are tested, including linear techniques, polynomial-augmented penalised linear techniques, and ensemble methods, including forest tree and gradient-based tree regressors. For each model, the same targets (i.e., the VWC reference vales) are chosen, while different sets of variables are used for the training:

1. Raw readings only;
2. RSSIs only;
3. Raw readings together with RSSIs.

In so doing, each of the ML models has three versions for the functions of the exploited training data. In particular, the first version only processes raw readings in order to map them into VWC estimates to obtain a calibrated version of the real soil moisture sensor, the second one describes a relationship between RSSIs and VWCs aiming at setting up a virtual sensor for VWCs, while the third estimates VWCs simultaneously accounting for both soil moisture sensor raw readings and RSSIs, resulting in a full-fledged augmented sensor for measuring VWC. The considered ML models are the following: ordinary least squares (OLS) regressor, ridge regressor, least absolute shrinkage and selection operator (LASSO) regressor, logistic regressor, support vector machine (SVM) regressor, random forest (RF) regressor, gradient boost (GB) regressor and extreme gradient boosting (XGBoost) regressor. Such models are taken into account because they are the most adopted for regression purposes.

Data was divided into training and test sets (i.e., 70% and 30% of the collected dataset, respectively) keeping an equal balance between different VWC reference values in the training and test sets. Moreover, in order to improve performances, a hyperparameter optimisation procedure was applied by resorting to a k-fold cross-validation approach with five non-overlapping folds. Indeed, the performances of each ML model depend on its hyperparameters. For instance, in the case of ensemble tree methods, some of them are the number of trees and their maximum depth, or the learning rate that shrinks the contribution of each tree. For each model, the values of relative hyperparameters were chosen by dividing the training set into training and validation subsets and exploring performances from values of the hyperparameters spanning in a grid (i.e., a grid-search approach). Finally, the root mean squared error (RMSE) was exploited as a loss function during the training phase.

## 6. Results and Discussion

### 6.1. Machine Learning Model Selection

Table 2 and Table 3 report the performance parameters of the tested ML models, respectively, for the training and test sets. They summarise mean estimation error $\mu_{e}$, root mean square estimation error $\sigma_{e}$, and the width of the VWC intervals containing, respectively, 75% (i.e., $w_{75}$) and 98% (i.e., $w_{98}$) of the estimation errors.

The consistency between the training and test procedures, as well as the absence of overfitting during training, arises by comparing the values of Table 2 and of Table 3. Moreover, the ensemble methods turned out to work better and provided similar performances. As a matter of fact, ML models implementing virtual sensors (i.e., the ones based only on RSSIs) gave the worst performances. This result directly stems from the discussion about Figure 7b, reflecting the lack of a bijective relationship between RSSIs and VWCs. On the other hand, the best performances are ensured by the ML models carrying out augmented sensors (i.e., the ones resorting to both soil moisture sensor raw readings together with the RSSIs). Finally, the ML models taking as input just the sensor raw readings showed medium-quality performances. This is because any model based only on moisture sensor raw readings can be viewed as the implementation of a calibration procedure aiming at improving the capabilities of a coarse sensor device that is just able to distinguish between “wet” and “dry” states of the soil.

**Table 3:** Performances of analysed ML models for the test set. $\mu_{e}$ is the mean estimation error, $\sigma_{e}$ the root mean square estimation error, $w_{75}$ and $w_{98}$ are the width of the VWC intervals containing, respectively, 75% and 98% of the estimation errors. The first set of columns presents the performances of the model versions grounded on raw readings only (i.e., the calibrated real sensors). The second set of columns presents the performances of the model versions relying on RSSIs only (i.e., the virtual sensors). Finally, the last set of columns presents the performances of the model versions accounting for both the raw readings and RSSIs (i.e., the augmented sensors).

	Calibrated Real Sensors				Virtual Sensors				Augmented Sensors			
	Raw Reading				RSSI				Raw Reading + RSSI			
Model	$\mu_{e}$	$\sigma_{e}$	$w_{75}$	$w_{98}$	$\mu_{e}$	$\sigma_{e}$	$w_{75}$	$w_{98}$	$\mu_{e}$	$\sigma_{e}$	$w_{75}$	$w_{98}$
OLS	0.00	7.20	7.76	28.62	−0.09	9.67	17.83	33.84	−0.02	6.23	7.30	22.25
Ridge	0.00	7.20	7.76	28.62	−0.09	9.67	17.91	33.83	−0.02	6.23	7.30	22.25
LASSO	0.00	7.17	7.37	28.66	−0.09	9.63	17.81	33.60	−0.01	6.23	7.59	22.29
Logistic	1.14	4.40	0.00	20.00	0.84	11.00	10.00	45.00	−0.10	2.10	0.00	10.00
SVM	1.31	4.83	3.63	25.63	−0.72	9.29	11.14	37.90	0.04	2.12	0.20	9.80
RF	0.04	2.91	3.31	11.12	−0.05	8.93	11.47	37.04	0.01	1.43	0.01	7.12
GB	0.03	2.91	3.50	11.91	−0.04	8.93	11.54	36.82	0.01	1.43	0.13	7.13
XGBoost	0.02	2.91	3.57	11.90	−0.05	8.93	11.51	36.90	0.01	1.42	0.08	7.13

Generally, the ensemble tree methods ensured satisfactory results [54]. Indeed, as shown in Table 3, the XGBoost model proved to be the best as it provided the best metrics. In particular, the relative augmented sensor (i.e., the one taking as input both the RSSIs and raw readings) improved the model based only on the soil moisture sensor raw readings by approximately three times. Therefore, in light of this, and because of the computational efficiency of XGBoost, it is selected as the candidate model to address VWCs estimation. Accordingly, the following discussion only accounts for such a model.

### 6.2. XGBoost Regressor Performances

**Figure 9:**
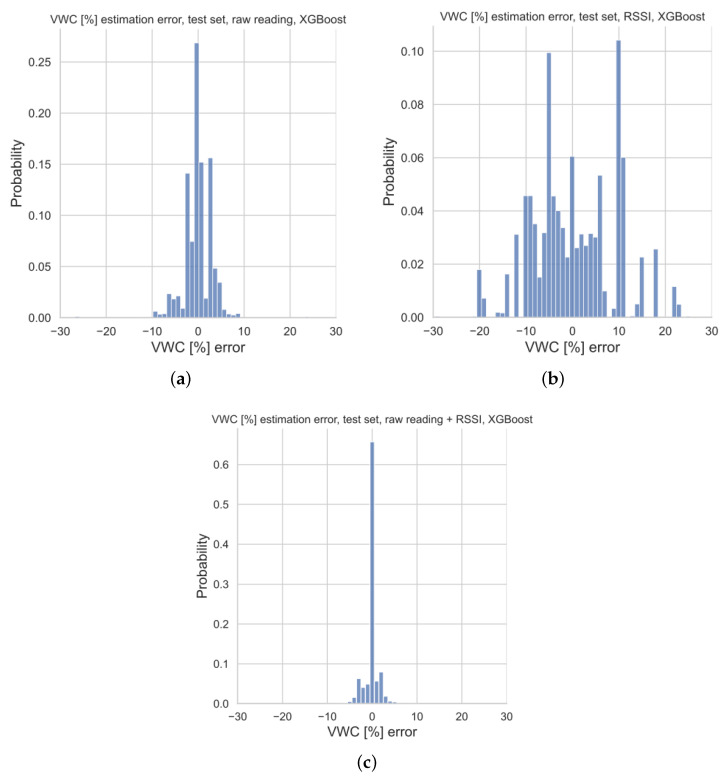
Histograms of the estimation errors of the predicted VWC values for the three model versions on the test set: (**a**) calibrated real sensor relying only on raw readings, (**b**) virtual sensor taking as input RSSIs and (**c**) augmented sensor grounded on both raw readings and RSSIs.

Histograms of regression errors for each of the three model versions are provided in Figure 9. The version resulting from the raw readings (i.e., the calibrated real sensor) provides errors ranging from approximately ±10% of the estimated VWC. On the other hand, the regression error related to the model version implementing the virtual sensor spans ±20% of the estimated VWC. Finally, the augmented sensor entails an error in estimates which is far narrower (i.e., ±8%), being slightly more than the quantization step of the VWC reference values.

**Figure 10:**
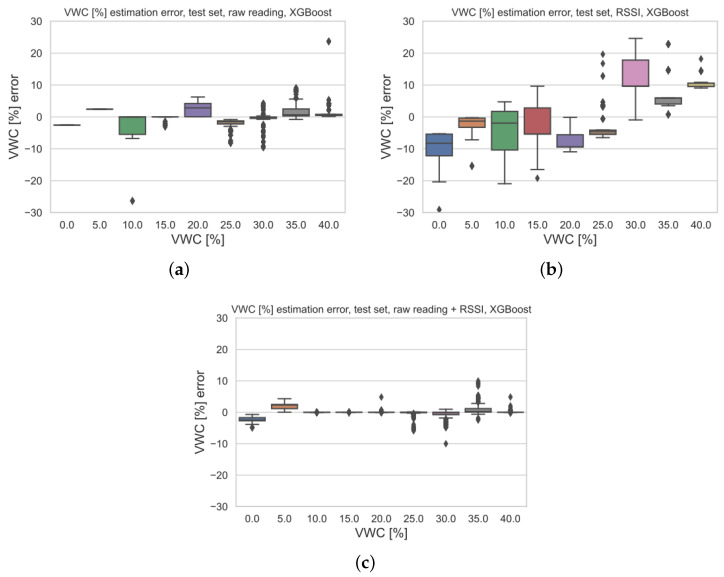
VWC estimation errors on test set for the three model versions: (**a**) calibrated real sensor relying only on raw readings, (**b**) virtual sensor taking as input RSSIs, and (**c**) augmented sensor grounded on both raw readings and RSSIs. Each colour in the picture stands for a different level of VWC.

The analysis of possible relationships between the estimation errors and VWCs for each of the model versions is given in Figure 10 by means of standard boxplots associated with the distribution of regression errors for all of the VWC reference values. Each of the boxplots shows the minimum, the maximum, the sample median, and the first and third quartiles for a given reference value of VWC, along with relative outliers. Moreover, in order to enhance the comparison, the same vertical scale was adopted for all the boxplots. The boxplot for VWC =10% of Figure 10a displays a case where the sample median error is close to 0%, while the errors are within a range of [−5%, +3%] within a 50% probability (i.e., the 1st quartile). Conversely, the other boxplots of Figure 10a show that the calibrated real sensor (i.e., the one only relying on soil moisture sensor raw readings) is affected by a rather large bias effect superimposed on the random error contributions. This shortcoming is largely reduced for the augmented sensor (see Figure 10c), where the median error is 0% in almost all of the cases regardless of the VWC, and barely no bias is experienced, implying strong robustness due to the simultaneous usage of both the RSSIs and the raw readings. Finally, the estimation error for the virtual sensor (i.e., the one taking as input just the RSSIs) is highly susceptible to VWC (see Figure 10c) due to the large relative bias. Indeed, as previously explained, this model version is the one resulting in the worst performances.

The virtual sensor exploiting only the RSSIs is able to provide rough VWC estimates because its RMSE is 8.67%; this is predictable since water alters radio propagation. Conversely, the calibrated real sensor improves the quality of the estimates providing an RMSE of 3.33%. Moreover, it enables the low-cost, low-precision soil moisture sensor to distinguish VWCs as an actual sensor, rather than to discriminate between “wet” and “dry” states of the soil. This is a remarkable result, showing that ML methods may considerably improve the performances of a very rough sensor. Finally, the combination of raw sensor readings and RSSIs, resulting in the augmented sensor, boosts the performance estimates ensuring an RMSE of 1.84%.

**Figure 11:**
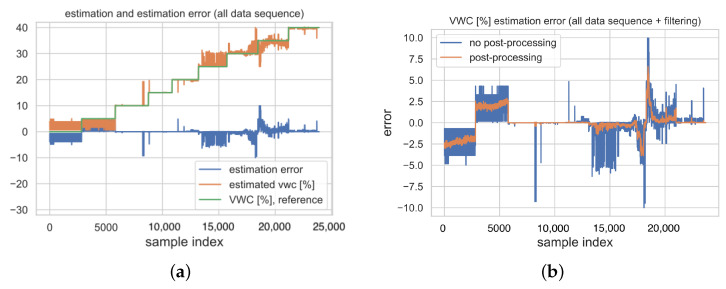
Effect of data post-processing (i.e., Savitzky–Golay filtering) for the augmented sensor (i.e., the one taking as input both raw readings and RSSIs) on the test set: (**a**) sequence of VWC estimates and estimation errors for the unfiltered model, and (**b**) comparison between estimation errors with and without filtering.

In order to reduce the outliers (see Figure 10), and consequently enhance performances, post-processing techniques exploiting the random nature of such events can be applied. This fact is clearer in Figure 11, which shows the sequence of estimated VWCs as a time-series according to the acquired data during the dataset collecting procedures. Specifically, Figure 11a gathers the sequences of actual VWC estimates, reference VWCs and estimation errors. It can be seen that, at higher VWCs, more outliers can be found, as confirmed by the boxplots. For this reason, a simple Savitzky–Golay filter of the first order was applied [55]. Of course, the higher the filter order (e.g., 1000), the better the reduction in spurious peaks, but this may imply that measurements will suffer from high processing overheads and considerable latency. Therefore, a reasonable trade-off consists in adopting a mild filtering window, so that isolated peaks are cancelled out, while more frequently repeated ones are just reduced. Indeed, the plot in Figure 11b shows the corresponding outcome for a width of the filter equal to 51.

**Figure 12:**
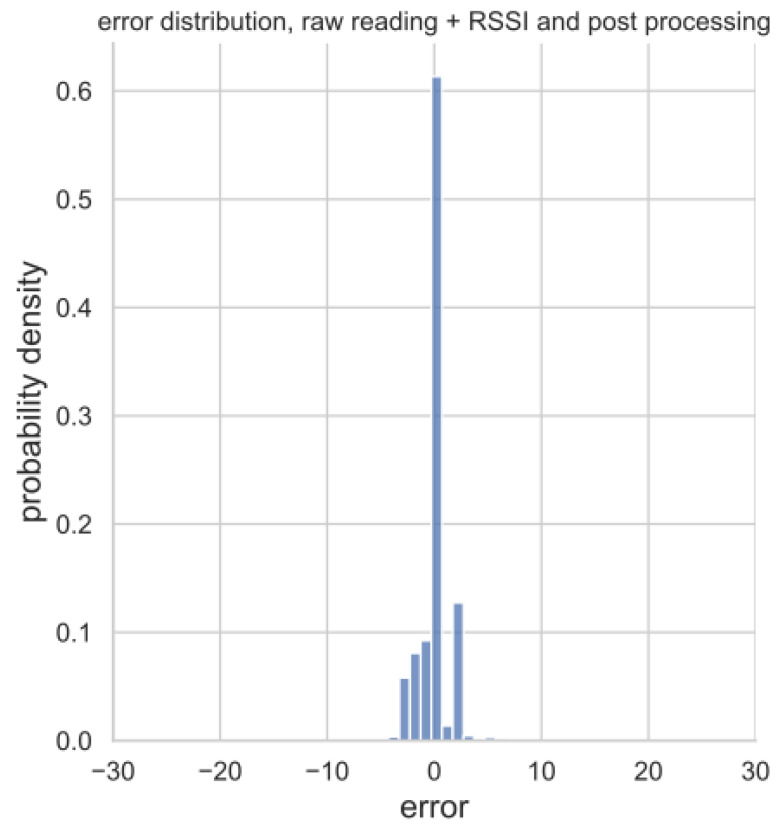
Histogram of estimation errors for the augmented sensor (i.e., the one taking as input both raw readings and RSSIs) on the test set after filtering.

Consequentially, with regard to the augmented sensor (i.e., the one taking as input both raw readings and RSSIs), filtering enhances the estimation error probability (see Figure 12), and reduces the RMSE down to 1.53%. Specifically, from Figure 12, the estimation error strictly ranges within −5% and +5%, thus becoming comparable to the adopted quantization step for VWCs during the measurement campaign for the dataset gathering.

Finally, factors such as burial depth, soil composition or $L_{AG}$ were not explored in the experiments reported since they can be considered and compensated within calibration phases of the estimation algorithms.

### 6.3. Comparison with Related Work

As stated in Section 2, no related studies presenting a similar approach with respect to the one of this paper were found. Indeed, many contributions highlighted the relationship between the dielectric properties of soil, mainly depending on VWC, and wireless links quality indicators (e.g., RSSI), but such correlations were not exploited to perform inverse sensing of VWC [14,15,16,17,18,19,20,21,22]. On the other hand, other studies set up systems for inverse sensing of VWC [23,24,25], although such solutions were characterised by system infrastructures and sensing techniques which are much more complicated and expensive than the one proposed in this paper. Some research also involved the development of systems for the estimation of VWC. For instance [26], though no field tests were performed, and [27,28,37], that exploited GPR techniques, and [29], that resorted to satellite imagery. However, GPR instrumentation is quite expensive; thus, it is not suited to the deployment of a pervasive network infrastructure needed for precision agriculture. On the other hand, satellite imagery can only be exploited to sense superficial soil moisture, and only when such data is available, translating into a low sampling rate. ML techniques have been exploited for inverse sensing of VWC [33,34,35,36], but tackling the problem from a different perspective than the one of this paper. Such solutions predicted the future values of soil moisture content, rather than in real-time, as can be done by exploiting the findings of this paper. Additionally, the ML-enabled methods did not make use of radio transmission parameters, such as RSSIs, as additional data for the improvement of ML algorithm performance.

The main limitation of the proposed approach is common to any data-driven method. As the proposed methodologies are grounded in data collection strictly dependent on the soil topology, they necessarily require a starting stage during which they have to undergo a setup and tuning step, implying the collection of additional data related to the soil at hand. Nonetheless, all of the proposed techniques (i.e., the calibrated real sensor, the virtual sensor, and the augmented sensor) can be replicated for whichever soil composition, highlighting the methodology adaptability.

## 7. Conclusions

The aim of this paper was to propose the usage of ML-based techniques to improve the accuracy of LoRaWAN-based wireless soil-moisture-sensing systems, aiming at measuring VWC. In particular, the relation among VWC and RSSIs was exploited in two ways: firstly, by setting up a virtual sensor obtaining a rough VWC estimation by only exploiting RSSIs; secondly, by combining these values with measurements acquired with a low-cost, low-precision soil moisture sensor, thus achieving an augmented sensor. The proposed method was assessed by means of a measurement campaign aimed at collecting a real dataset containing both RSSIs related to UG2AG LoRa links, and the low-cost, coarse soil moisture sensor readings as a function of several levels of VWC. Such tests were devised in a controlled experimental environment by resorting to a plastic case filled with sand. It was gradually watered to augment the VWC, while LoRaWAN RSSIs and the raw sensor readings were regularly acquired. In so doing, nine levels of VWC were obtained, during each of which three transmission batches of 1000 LoRaWAN packets were executed.

Acquired data was used to evaluate the performances of different ML regression algorithms for the estimation of VWCs using three different sets of variables, leading to the identification of an ensemble-tree-based algorithm as the best model for the specific target (i.e., XGBoost). In the first case, only raw readings coming from the coarse sensors are exploited, leading to a model implementing the calibrated version of the real sensor. Then, only RSSIs are used, thus obtaining a virtual sensor. Finally, both RSSIs and raw readings from the soil moisture sensors are employed, turning the relative model into a full-fledged augmented sensor. The results demonstrated that the virtual sensor is able to roughly estimate the VWC level, the calibrated real sensor gives intermediate performances, while the augmented sensor provides the best VWC estimates. Moreover, the augmented sensor can be further improved by applying a post-processing filtering technique (i.e., the Savitzky–Golay filter), ensuring an estimation error which is comparable to the adopted quantization step for VWCs during the measurement campaign for the dataset gathering.

The results of this paper can be of significant importance for the actual deployment of distributed measurement systems in application scenarios which are typical of precision agriculture contexts. Indeed, a large number of connected monitoring devices is generally required to cover the large surfaces of cultivation and crops. The availability of low-cost devices and accurate methods may boost the adoption of intelligent systems, with notable benefits for overall reduction in the environmental impact of industrial crops. Nonetheless, this paper’s findings can be easily applied wherever WUSN are concerned.

The current limitations of the proposed methodology are those that any data-driven approach implicitly entails. Indeed, because the proposed sensing techniques are based on a dataset gathering information related to a single typology of soil (i.e., pure sand), extending them to any soil composition may require an initial setup and tuning stage needing the collection of additional data related to the soil at hand. However, this also means that the presented methodology can be fully repeated for whichever soil composition is considered. Nonetheless, in future work, provided there is accurate soil analysis providing its composition, the proposed methodology can be easily improved by injecting in the models the knowledge coming from the theoretical analysis explained in Section 3. In so doing, the overall robustness and reliability of the proposed sensing techniques will be notably enhanced.

## Figures and Tables

**Table 1:** Related works key findings summary.

Reference Number	Authors	Year	Key Findings
[12]	Parri et al.	2020	The robustness and reliability of the LoRaWAN protocol was proven by means of a 70-day test period during which offshore wireless links covering 8.33 km were validated.
[13]	Abrardo et al.	2019	The feasibility of multi-hop LoRa networks was studied bearing in mind the application scenario of underground medieval aqueducts monitoring.
[14]	Lin et al.	2020	The link quality of LoRa WUSN was analysed showing that semi-empirical path-loss models were successfully verified by means of field tests, demonstrating that the communication range can be greater than 50 m at the burial depth of 0.4 m by adjusting the LoRa transmission/receiving settings.
[15]	Silva et al.	2015	Attenuation properties of soil in WUSN were modelled and preliminarily validated by means of experiments (both indoor and outdoor) involving a magnetic-induction-based WUSN.
[16]	Duisterwinkel et al.	2018	A system made of underground sensor nodes for the remote monitoring of underground water pipeline was proposed, hinting at the potential feasibility of a WUSN for such an application.
[17]	Di Renzone et al.	2021	Theoretical path-loss models for UG2AG LoRaWAN links were validated and compared with results of field tests involving three types of soils and several burial depths, showing a discrepancy between measurements and estimates coming from models, while validating the robustness of the LoRaWAN protocol in such application scenarios.
[18]	Mejjatti et al.	2017	The behaviour of a dipole antenna, operating in the UHF band, buried within wet soil was analysed by means of simulations, showing an inverse relationships between VWC, operating frequency and bandwidth, leading to a modification of the antenna radiation diagram, making it more directive.
[19]	Trang et al.	2016	The dependence of wireless transmission performances within sub-GHz spectrum in the function of soil characteristics was studied exploiting simulations.
[20]	Malik et al.	2018	The dependence on soil moisture of transmission performances for LoRa underground links was investigated by means of simulations, and by comparing the results with the ones stemming from links enabled by cellular technologies, such as NB-IoT
[21]	Lombardo et al.	2021	The robustness and feasibility of LoRaWAN and NB-IoT were validated for critical contexts via field tests involving transmissions from underground, underwater, and through metallic enclosures.
[22]	Horvat et al.	2016	The specific effects of soil on LoRa systems were studied by accounting for the correlation between performance drops, inter-nodal distance and soil moisture.
[23]	Liedmann et al.	2017	An innovative multidimensional radio-field-based system for measuring soil moisture was developed, showing that variation in the underground communication link quality can be exploited for sensing soil moisture.
[24]	Liedmann et al.	2018	The system developed in [23] was enabled by LoRa modulation and tested in the field for long-term evaluation of soil moisture, showing that RSSIs were suitable for such an application.
[25]	Jiang et al.	2019	A fully customised hardware platform, including LoRa modules, was developed for smart farming contexts. The system was tested for six months by deploying sensor nodes in agricultural sites.
[26]	Di Fusco et al.	2018	The estimation of soil moisture by resorting to radio waves (i.e., LoRa) parameters was proposed from a theoretical point of view by means of a global sensitivity analysis, but no field tests were performed.
[27]	Zhou et al.	2019	A laboratory study on three soil specimens aimed at assessing the viability of high-frequency GPR antennas to sense the water content in soils was performed, confirming the crucial effect of dissolved water on soil attenuation properties.
[28]	Anbazhagan et al.	2020	A laboratory study on four soils was performed to measure water content by means of GPR instruments, confirming the dependence of soil attenuation properties on dissolved water.
[29]	Ainiwaer et al.	2020	Satellite imagery was exploited for estimating soil moisture content at a regional scale, thus suggesting an indirect method for sensing soil moisture.
[30]	Ali et al.	2015	Literature review on remote sensing of vegetation biomass and soil moisture exploiting machine learning methods.
[31]	Samie et al.	2019	Literature review on the role of ML in IoT from the cloud to embedded devices.
[32]	Ahmad et al.	2010	An ML model (i.e., support vector machine) for soil moisture estimation relying on remote-sensing data was proposed.
[33]	Adab et al.	2020	Several ML models inferring soil moisture on the basis of satellite imagery were devised and compared showing the potential feasibility of such an approach.
[34]	Hong et al.	2016	A methodology combining soil moisture measurements and meteorological data was proposed. It was able to predict soil moisture a day ahead, providing a useful tool for precision agriculture.
[35]	Matei et al.	2017	A system for predicting soil moisture on the basis of meteorological data and soil temperature data was set up and tested in the field to assess its accuracy.
[36]	Dubois et al.	2021	Soil moisture content was predicted by exploiting supervised learning algorithms taking as input several types of data (e.g., meteorological data related to temperature and rainfall). Then, the solution was tested for three years in potato farming to evaluate system performances.
[37]	Liang et al.	2017	ML architectures were devised to infer VWC from radio echoes generated by means of UWB GPR, achieving satisfactory results.

**Table 2:** Performances of analysed ML models for the training set. $\mu_{e}$ is the mean estimation error, $\sigma_{e}$ the root mean square estimation error, $w_{75}$ and $w_{98}$ are the width of the VWC intervals containing, respectively, 75% and 98% of the estimation errors. The first set of columns presents the performances of the model versions grounded on raw readings only (i.e., the calibrated real sensors). The second set of columns presents the performances of the model versions relying on RSSIs only (i.e., the virtual sensors). Finally, the last set of columns presents the performances of the model versions accounting for both raw readings and RSSIs (i.e., the augmented sensors).

	Calibrated Real Sensors				Virtual Sensors				Augmented Sensors			
	Raw Reading				RSSI				Raw Reading + RSSI			
Model	$\mu_{e}$	$\sigma_{e}$	$w_{75}$	$w_{98}$	$\mu_{e}$	$\sigma_{e}$	$w_{75}$	$w_{98}$	$\mu_{e}$	$\sigma_{e}$	$w_{75}$	$w_{98}$
OLS	0.00	7.20	7.64	28.77	0.00	9.66	18.11	33.84	0.00	6.23	7.20	22.30
Ridge	0.00	7.20	7.64	28.77	0.00	9.66	18.16	33.83	0.00	6.23	7.20	22.30
LASSO	0.00	7.18	7.25	28.80	0.00	9.63	18.18	33.75	0.00	6.23	7.38	22.29
Logistic	1.12	4.40	0.00	20.00	0.83	11.07	10.00	45.00	−0.11	2.13	0.00	10.00
SVM	1.31	4.88	3.65	26.65	−0.72	9.35	11.14	37.90	0.04	2.12	0.20	9.80
RF	0.00	3.04	3.30	11.71	−0.01	8.96	11.47	37.04	0.00	1.42	0.01	7.10
GB	0.00	3.03	3.50	11.94	0.00	8.96	11.54	36.82	0.00	1.42	0.11	6.89
XGBoost	−0.01	3.03	3.57	11.99	−0.01	8.96	11.51	36.9	0.00	1.41	0.08	6.75

## Data Availability

Data is available from the corresponding author upon request.
